# Narratives of Herbal Medicine Utilisation in the United Kingdom: Scoping Literature Review

**DOI:** 10.3389/fphar.2022.886574

**Published:** 2022-08-25

**Authors:** Celine Longden-Naufal, Vivien Rolfe, Marion Mackonochie

**Affiliations:** ^1^ Independent Researcher, London, United Kingdom; ^2^ Pukka Herbs Ltd., Keynsham, United Kingdom

**Keywords:** gender, herbal medicine, perceptions, thematic analysis, traditional medicine, United Kingdom, attitudes, ethnic communities

## Abstract

Using thematic analysis of existing literature, this scoping review aims to explore the narratives of people using herbal medicine (HM) in the United Kingdom. Understanding who is using HM and why will enable better ways of facilitating the use of HM, as well as assist in designing future research. Ethnic groups were found to be primary users of HM in the United Kingdom. A sense of heritage continues to be important for these participants as it allows tradition and culture to stay alive within communities, as well as the ritualistic purposes of these plants. For women, another key demographic, concepts surrounding the naturalness of HMs are associated with the idea of femineity and self-healing. A reoccurring theme in the literature focusing on both ethnic groups and women’s perceptions is the judgement from healthcare practitioners/professionals (HCPs) when addressing the use of HM. However, studies that investigated the perceptions of HCPs on HM confirmed that they often were supportive of using HM where standard treatments had been unsuccessful, and if a patient had anecdotal evidence of a herb having been effective. Delving deeper into public narratives of HM usage will allow conventional healthcare systems to effectively integrate alternative approaches, as well as ensuring that future research into the benefits of HMs is relevant to how people use them.

## 1 Introduction

In the United Kingdom, herbal medicine (HM) is a treatment modality that falls under the broad umbrella of complementary and alternative medicine (CAM). Whether to see a herbalist or purchase herbal supplements is a decision made by individuals as an addition or alternative to the National Health Service (NHS). The NHS is the publicly funded healthcare system in the United Kingdom and is free at the point of use. In contrast, HM must be sought out as a paid-for extra by individuals who want or need an alternative.

Globally, there are many studies on the clinical efficacy of medicinal plants, and through ethnopharmacological research, anthropologists continue to explore universal concepts in medicine, and the way that they are embodied through diverse health systems. HM involves the use of natural materials as medicines, however, approaches from traditional medicine also involve psychological, spiritual and social aspects (Jäger, 2015). One of the arguments against carrying out research into the efficacy of single plants or even poly-herb formulae is that this does not take into account these additional features of traditional medicine treatment that undoubtedly play into why people seek out HM, and these aspects may also relate to whether they are effective.

Traditional knowledge on the use of plants is being eroded due to a change in scientific focus and following less recognition by healthcare professionals (HCPs; Lazarou and Heinrich, 2019). However, some HCPs believe that HM should be used alongside standard medicines and patients should be encouraged to talk about any herbal treatments they may be using during a medical consultation (Zahn et al., 2019). Understanding if and how medicinal plants are effective and safe is important to HCPs but this may not be as important to the people who use them.

Since the COVID-19 outbreak, certain herbal extracts such as Andrographis (*Andrographis paniculata* (Burm.f.) Nees) and mushrooms like *Grifola frondosa*, *Agaricus blazei* Murrill and *Hericium erinaceus* have been clinically explored to help fight the virus with many promising results in international studies (Frost et al., 2021; Hetland et al., 2021). However, in the United Kingdom, there has been less research into the use of herbal treatments with only one HM trial of Sambucol Black Elderberry liquid running in the United Kingdom (Frost et al., 2021). This indicates a reluctance to embrace the use of HMs within the United Kingdom, despite an acknowledgement of the importance of self-care for health.

In the United Kingdom, there remains a lack of knowledge by patients and their healthcare providers on not just HM but in CAM, and for a more successful integrated healthcare system, there needs to be greater understanding of when CAM is appropriate for each individual patient (Akhtar and Wong, 2021:33; Bhamra et al., 2019). To ensure a smooth integration of alternative practices like the use of HM, HCPs need to gain a holistic comprehension of why these practices are increasingly being used. Most of the available literature use quantitative data collection and analysis that only grazes the surface of emerging narratives; qualitative research generally focuses on either a singular narrative or a particular population group. This study aims to probe into the core narratives surrounding HM in the UK from multiple studies to understand the reasons behind its use and identify areas where further research is necessary.

## 2 Methods

A literature search was carried out, and a thematic analysis used to identify and analyse patterns and narratives within the research (Braun and Clarke, 2006). An analysis contextualising the population groups was carried out to aid the extraction of different narratives surrounding perceptions of HM in the United Kingdom.

A comprehensive search on PubMed was conducted using search phrases such as “Herbal Medicine”, “Medicinal Plants”, “Attitudes”, “Perceptions”, “UK”, “United Kingdom”*.* Google Scholar was also used to check for grey literature. No date restriction was implemented to ensure that relevant papers were not missed and to enable future analysis of possible changing perceptions over time. The search was limited to title, abstract and listed keywords. Further papers were found by manually searching through the bibliographies of retrieved papers. It should be noted that the strategy does carry some limitations due to searching only two bibliographic databases and stopping the sift through studies after less relevant papers started to be retrieved. There may have been relevant articles down the search list (Sodhi and Tang, 2017). The results of the search initially included literature focusing on the perceptions of CAM which resulted in 59 articles. However, as this article was to focus on the perceptions of HM, articles that did not include details and/or analysis of the perceptions of HM were excluded (Table 1), leaving 40 relevant studies (See Supplementary Table S1).

**TABLE 1:** Inclusion and exclusion criteria.

Inclusion Criteria	Exclusion Criteria
• Reporting perceptions and attitudes of herbal medicine/medicinal plants in the United Kingdom	• Not conducted in the United Kingdom
• Papers from medical/biomedical/biological journals	• Efficacy studies
• Papers from social science journals	• Studies on CAM that don’t include detailed discussion and results regarding HM
• Comparisons between the United Kingdom and other countries	• Literature reviews
• Studies that focus on CAM that includes detailed discussion and results regarding HM.	

Numerical codes were manually generated for identified study aims, data collection methodologies, locations and study populations which were then entered into an Excel spreadsheet (see Supplementary Table S2).

**FIGURE 1:**
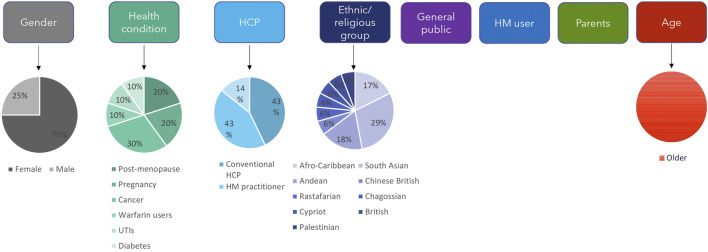
Key population themes and sub-themes. The different population themes for the studies are shown along the top. Age, gender, health condition, HCP and ethnic/religious group are further divided into sub-themes; shown by the pie charts below them that indicate the relative numbers of studies for each sub-theme. HCP: healthcare professional, HM: herbal medicine, UTIs: urinary tract infections.

The main themes of interest that emerged from the literature were the population groups of the study. By focusing on the population groups and their sub-groups (Figure 1), the study aims were more contextualised (i.e., population = has a health condition, sub-groups = post-menopausal, study aim = perception of HM for health conditions). Due to significantly lower numbers of papers associated with most of the populations, themes with less than five papers were excluded from further analysis as they would be unreliably represented. Those sub-categories are understudied themes that could be considered in future research. The majority of the sub-groups also consisted of five or less papers, therefore the analysis presented will not represent the general views of particular populations but can be used to lay a theoretical foundation based on the literature.

It was common for articles to be associated with multiple categories which explains considerably high numbers of articles per categories. The study aims “use of HM” and “perceptions of HM” could be considered as interchangeable, however it was decided to keep them as separate categories since multiple articles determined the use of HM and excluded the perception of them, and vice versa.

## 3 Results and Discussion

**FIGURE 2:**
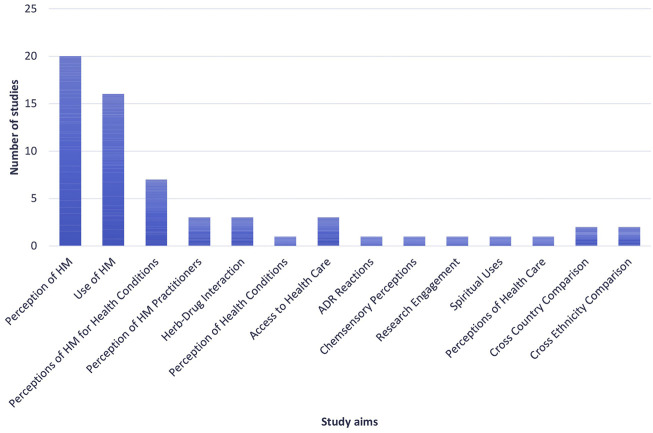
Number of studies allocated to each of the identified study aims. ADR: adverse drug reaction, HM: herbal medicine.

Within the retrieved papers, there were several clear aims, the two main ones being understanding the use of HMs and understanding the perceptions of HM use (Figure 2).

The discrete population groups identified were gender, ethnic/religious groups, those with specific health conditions, HCPs, general public, age, HM users and parents (Figure 1). The two population themes with five or more papers addressing them were explorations of gender and ethnic groups. Due to the qualitative nature of papers focusing on these two groups, as well as being the most frequently researched, there was more scope for constructing narratives from these studies, but it did not rule out papers that studied other populations.

### 3.1 Population Theme 1: Ethnic Group Narratives in the United Kingdom

From the literature, the primary populations to be studied in the UK were non-white ethnicities and migrant experiences, particularly those of South-East Asian, Andean and Afro-Caribbean descent (Table 2). The reason for a significantly higher number of studies focusing on these populations may be due to the higher levels of HM usage compared to those of British white ethnic descent, or that social science researchers may subconsciously be specifically targeting these populations through the “noble savage” rhetoric (Tupper, 2009). Nevertheless, from these groups, a focal and recurrent narrative was how using HMs channels a sense of identity amongst participants. Within the theme of ethnic minority populations in the United Kingdom, research on South Asian populations in the UK were most frequent (*n* = 5).

**TABLE 2:** Study aims for the studies associated with the population theme of ethnic/religious group.

Ethnic/religious group	Number of studies	Study aims
Afro-Caribbean	3	Use of herbal medicine
		Herb/drug interactions
		Perceptions of healthcare
		Cross-country comparison
		Research engagement
South Asian	5	Perception of herbal medicine
		Use of herbal medicine
		Chemosensory perceptions
		Cross ethnicity comparison
Andean	1	Perception of herbal medicine
		Use of herbal medicine
		Access to health care
Chinese British	1	Perception of herbal medicine
		Use of herbal medicine
Rastafarian	1	Spiritual uses
Chagossian	1	Perception of herbal medicine
		Cross country comparison
Cypriot	1	Perception of herbal medicine
		Use of herbal medicine
British	1	Cross ethnicity comparison
		Chemosensory perceptions
Palestinian	1	Access to health care

A common factor behind migrant experiences of HM use in the UK is to preserve culture. Jeffery’s ethnographic research on plant use in the Chagossian community in the United Kingdom, Mauritius and Seychelles explored the use of plants in those who are displaced from their homes and are experiencing social and cultural disruption (2016). Once in the UK, some of the community continued to use HMs but lack of access to medicinal plants affected use. Another concern raised was that younger generations of people are not exposed to knowledge of traditional medicines which causes a lack of interest—“displacement and migration can result in the transformation or loss of certain cultural heritage practices, including those involving plants” (Jeffery and Rotter, 2016: 303).

This study observed that when members of the Chagossian community returned home, they would often bring medicinal plants back for their family and friends. One older Chagossian woman observed that her family were reticent to take her bitter herbal tonics as they had more faith in doctors, although they enjoyed the sweeter taste of her mint and barley remedies. The author suggests that biomedicine removes healthcare from social relations, however, does not prevent all forms of healthcare as some are still embedded in social practices (Jeffery and Rotter, 2016: 306).

This is also identified in Bhamra et al.’s exploration of the use of HMs by diasporic South Asian communities in the UK (2017). Most older participants agreed they would talk to their family for advice on minor ailments, but younger people were less likely to as they were less aware of their traditional roots and traditional family knowledge (Bhamra et al., 2017). This issue was prominent in a 2005 analysis of the use of botanicals by the Sikh community in London. When older participants were asked what they thought about younger generations being less familiar with traditional knowledge about medicinal plants, Western influences and lifestyles were commonly seen as the primary cause for this loss (Sandhu and Heinrich, 2005).

In a further exploration of health-related decisions amongst older Ghanaian men, the concept of heritage was actually a reason as to why UK-based participants chose not to use HMs. This was partly due to the lack of availability of preferred herbs (also identified in Pieroni et al., 2008; Jeffery and Rotter, 2016; Ceuterick et al., 2008), but also because it was more difficult to identify a trusted practitioner—“*I used herbal mixtures in Ghana but, ever since I have been here (UK) I have not mainly because I cannot get some and also, even if I do, I do not know who made it. Perhaps if I get the original herbal mixtures here in the UK, I will gladly use them.*” (Alidu and Grunfeld, 2020: 6).

Nevertheless, in other UK communities there is still a vast wealth of traditional HM knowledge, for example South Asian populations. In Pieroni and Torry’s research of cross-ethnic chemosensory perceptions of the relationship between taste and medicinal properties, traditional knowledge of HM was higher in South Asian people compared to participants of English ethnicity residing in Bradford (2007). The Kashmiri population were most likely to perceive that there were medicinal benefits to herbs (i.e., ginger for infections and muscular disorders, mint for digestive and respiratory troubles etc.), closely followed by the Gujarati population. Pieroni et al. further explore the use of traditional HM by South Asian communities in West Yorkshire, focussing on Pakistani migrants from Mirpur (2008). Reflecting on the previous study, it wasn’t surprising that the majority of participants were more amenable to herbal remedies as they were more used to them being utilised by mothers and grandmothers in their households (Pieroni et al., 2008). When asked, these participants could list many medicinal plants that were still being used within their homes.

One study focused on the Andean communities in London, and the traditional use of herbs had declined because the conditions they were used to treat were less frequent; plant species used for the treatment of intestinal worms or other parasites were identified in the literature but were no longer needed by communities in London (Ceuterick et al., 2008). A widely recorded category of HM contained *tranquilizantes* (tranquilisers), *algo para dormir mejor* (sedatives) and *para dar animo* (anti-depressants), suggesting that stress and anxiety were common in this community, and that these herbs were being used as remedies either because the conditions were viewed as minor or that they were taboo (Ceuterick et al., 2008). This research highlighted that the use of herbs for mental wellbeing and health was not often detected in the literature, but the work provided an important view of mental health conditions experienced by immigrants and ethnic minority populations living in the United Kingdom. Understanding these areas are particularly important for many immigrants who experience post-traumatic stress disorder or who are experiencing challenging living standards (Ceuterick et al., 2008).

Elsewhere in the literature, herbs were experienced as valuable and intrinsic to people’s culture and tradition; the sensorial aspects and perhaps aromas of herbs provoked memories of home and became symbolic: “*If a grandmother passes it on to her daughter, that daughter to her daughter, and if it goes on like that for generations in a row, then it must have an effect. They tell this to you for a reason”* (Ceuterick and Vandebroek, 2017: 9).

Through intensified globalisation, the interchange between local and global pharmacopoeias is constantly accelerating, especially via media platforms and international trade (Leonti and Casu, 2013). Cross-cultural studies in the scientific world attempt to identify “universal and general patterns of human culture in space and time” (Leonti and Weckerle, 2015). By comprehending both the pharmacological and cross-cultural significance of botanical materials, ethnopharmacology does not only unravel a plant holistically, but will further understand how migrating populations adapt to various healthcare practices.

#### 3.1.1 Sub-Theme Within Ethnic Group Narratives: Cultural Sensitivity Within UK Healthcare

Understanding individual needs and those of the population are important for creating healthcare systems that are inclusive (Bhopal, 2012). An example of this is diabetes amongst migrants from South Asia living in the United Kingdom; studies have shown that these communities have poorer health outcomes when they migrate to the west (Kumar et al., 2016).

Pieroni et al. (2008) observed that some communities use karela (bitter melon, *Momordica charantia*) to help with diabetes (Pieroni et al., 2008). Similarly, the use of karela by Indian and Pakistani migrants in the UK was causing patients to decrease their standard medication and was provoking an unwillingness to adhere to what their HCPs were telling them: “*Herbal medications are not recognised in this country; like garlic and onion. In the past they said they were bad, they have done studies and now they say is good. They don’t study herbal medicine. Why? If people studied them then people would start using them since that’s the way treatments are officially recognised here”* (Porqueddu, 2017).

A crucial observation was that participants rarely discuss such self-care practices with their medical doctor due to the lack of approval and testing of herbal medicines. This was found by Bhamra et al. (2017) who explored the use of traditional HM by South Asian communities in Leicester and Birmingham. When participants who took HM alongside their prescribed medication were asked if they shared this information with their HCP, 69% did not tell their doctor whilst 82% did not tell their pharmacist (Bhamra et al., 2017). Similarly to Porqueddu’s research, these individuals were fearful of being treated differently or not receiving any treatment at all if they divulged they were using HMs to treat their conditions.

The potential for herbs and drugs to interact are recognised as a safety risk, with interactions from commonly available materials like ginseng and warfarin, or garlic and aspirin an area for concern (Agbabiaka et al., 2018). In the case of the UK’s South Asian population, it is possible that a lack of cultural awareness from HCPs is preventing patients from sharing vital information which could increase the risk of adverse events relating to drug-herb interactions. Cultural insensitivity in a medical setting is regularly observed and critiqued, highlighting the benefit of integrating a medical anthropological lens when understanding research engagement. Tom et al. found that use of HM by older Afro-Caribbean men was preventing men from participating in prostate cancer research (2016). A lack of cultural awareness meant that these men were underrepresented in research studies.

Using HMs as a coping strategy by Andean migrants in London was emphasised in another socio-economic context through Ceuterick and Vandebroek’s research; this examined how personal preferences for HMs can shape identities in Andean communities in the UK (2017). For some Andean participants, the personal use of HMs resulted from a lack of appropriate healthcare choice:

*“It is always paracetamol, for everything. It probably helps, but I just do not take it anymore, because one person goes to a doctor with a certain ailment and gets a prescription for paracetamol, another person with another ailment will have to take paracetamol as well. I just do not have a lot of faith anymore.”* (Ceuterick and Vandebroek, 2017: 16).

Ceuterick and Vandebroek argue that using HM as a coping strategy for Andean migrants becomes part of the existing narrative about experiences with the NHS, and that the use of HM can be the result of a lack of positive choices (2017). It should be emphasised that the other reasons for HM use in this study do not solely focus on the perceived failures of the British healthcare system. HMs were also commonly used to keep tradition alive, a way to cope with the struggles of migration, and a symbol of home by Andean communities in London. For other HM users, there was a risk of being identified as “poor and uneducated” in the context of using HM for rituals in the UK, resulting in these participants disassociating themselves with these practices (Ceuterick and Vandebroek, 2017).

In contrast to exploring patient perceptions, other studies have looked at the use of HM by HCPs from different ethnic backgrounds (Bhamra et al., 2019). Over a third of participants in the study used HMs, and professionals from South Asia were most likely as their family backgrounds provided them with a degree of knowledge. This study also observed that professionals were more willing to learn about HMs that had been researched and where there was an evidence base to support their use. Others suggested there was not enough training on HMs partly due to medical curricula already being congested, a lack of funding and lack of staff interest (Bhamra et al., 2019). Providing HCPs with training and education on the use of HMs would improve patient communications and cultural understanding.

### 3.2 Population Theme 2: Herbal Medicine and Gender

Ten studies compared HM use between males and females in the UK as a part of their descriptive data—eight concluded that females were the predominant users of HM whilst two indicated that there was no significant difference. From the seven studies that focused on a particular gender, females were most frequently researched regarding their perceptions of HM (*n* = 5).

Throughout the 20th century, models of human evolution stemmed from the concept of Man, the Hunter, where male hunting was deemed its driving force. This evidently shed a light on a typically neglected area of study in the 1980s: Woman, the Gatherer, highlighting the importance of a woman’s role in hunter-gathering society, in particular as cultivators and medical users of medicinal plants (Howard, 2001; Fitzpatrick and Berbesque, 2018). Ethnobotanist Patricia Howard concluded that “*women predominate in plant use and management as herbalists, seed custodians and plant breeders, and gender relations universally have profound effects on how people and plants interrelate”* (2001: 17). Human evolutionary research can therefore suggest a reason for why women may have been more likely to be involved in healing and use of plants in the past (Howard, 2001: 34), but can it explain why females are currently the predominant users of HM in the Western world?

#### 3.2.1 Sub-Theme Within Herbal Medicine and Gender: More Judgement Within the UK Healthcare System

Perceptions of HM in relation to being female was a common topic found in the literature. There are a variety of HMs available in the UK to aid with fertility, pregnancy and post-partum health. However, Holst et al. (2009) observed that little research has been published about the motivations for using herbs to support female health. As with herbal medicine users from different ethnic groups, a reoccurring theme in the literature focusing on women’s perceptions is the judgement from HCPs about the use of HMs. All the participants in the study by Holst suggested that the NHS could be more accepting of the use of herbs because it is something that their patients are already using, and that professionals should have more knowledge of alternative treatments (Holst et al., 2009). They reported that HCPs were negative or dismissive of the use of HM, and in some cases, participants felt ridiculed for asking. There is an underlying issue relating to the lack of availability of information from HCPs to support individuals in their decision-making. Whether this means that HCPs need to provide more inclusive communication or HCPs need further training in HMs needs further clarification.

In a second study on the use of CAM and HM by women during pregnancy, most women participating were also reluctant to mention their use of HM to either their general practitioner (GP) or midwives due to the fear of being judged or because they were aware of the HCPs lack of knowledge on the subject. Instead, responsibility for using HMs was shifted from the HCP and onto the woman (Warriner et al., 2014). The women participating acted with more autonomy, which could be due to their need to feel confident and in control of their own health alongside their baby’s, suggesting that herbs play a role in empowerment and not just in benefitting health. Whilst these studies were in the context of pregnancy, there is less focus on how HMs relieve particular symptoms of pregnancy, labour, and postnatal discomfort. Instead, these narratives dissect a woman’s gendered existence in the British healthcare system.

Multiple studies have suggested that primary predictors of HM use in the UK are white women with higher educational status and higher income (Gokhale et al., 2003; Vickers et al., 2006; IPSOS Mori, 2008; Little, 2009; Gentry-Maharaj et al., 2015; Nissen, 2015). As seen with other patient groups, there was a lack of communication between patients and their HCPs regarding HM use. The majority of women in one study did not inform their doctors that they were using herbs, stating that there was no communication on the subject, that they felt the combination of herbs and prescribed medication is harmless, and that they feared the response from their doctors who lacked interest in the subject or were perceived to feel negatively about HM (Vickers, et al., 2006). The minority of women who informed their doctor stated that they have a good relationship with them and that they kept an open mind. One of those participants mentioned that her doctor was female—this suggests that a female doctor for a female patient might allow a more comfortable environment to discuss HM, or perhaps female doctors could be more open to HM?

Flower et al. (2015) investigated the attitudes towards HMs by GPs and the management of UTIs in Hampshire, and many were open about their lack of knowledge of HM which would hinder them from utilising herbal remedies. Several concerns were expressed including the lack of regulation, lack of quality assurance, lack of knowledge of herb-drug interactions and lack of clarity of the status of herbal practitioners (Flower et al., 2015). This is likely due to herbal practice being an unregulated profession in the UK, and that it is difficult to identify properly trained practitioners (Shaw et al., 2008). In the United Kingdom, several professional organisations exist including the National Institute of Medical Herbalists (NIMH), College of Practitioners of Phytotherapy (CPP), Complementary and Natural Healthcare Counsel (CNHC) and Association of Naturopathic Practitioners (ANP) who help maintain high standards of competence and support their membership. However, participation is voluntary:

*“Anyone can practise herbal medicine without the need for a licence or any qualifications. There are a number of voluntary registers which require that certain standards of practice and education are met, but these are not legally binding”* (Walker, 2015)*.*

Nevertheless, many GPs in one study were open to using herbs if conventional medicine was unsuccessful, if there was evidence supporting the use of herbs or if patients had anecdotal evidence of effective treatment (Flower et al., 2015).

#### 3.2.2 Sub-Theme Within Herbal Medicine and Gender: Who Says That Natural = Safe?

Herbal medicines and forms of CAM are rooted in historical and social associations with women’s practices of healing which hold connotations of “gentleness, goodness and safety” (Nissen, 2015). In Holst et al.’s (2009) study, the most common reason for using herbs that participants suggested was they were thought to be safer than pharmaceuticals, with one participant highlighting the length of time that humans have used HM. This is a comparable view to participants in other studies (Warriner et al., 2014). Even when some acknowledged that this did not always hold true, participants still felt herbs were generally safer than pharmaceuticals. There was also the recognition that more information needed to be distributed amongst patients (2014). The women in Vickers, Jolly and Greenfield’s study also addressed the safety of HMs, and that several female participants were not aware of possible herb-drug interactions as they had not experienced any adverse reactions themselves and felt that herbs were mild and good for you (Vickers et al., 2006).

Referring back to current UK regulations, “*anyone can, in the course of their business, make up, supply and administer herbal medicine, providing that they do so, on their own premises, that those premises can be secured, and a face-to-face consultation is carried out beforehand.”* (Walker, 2015). In Lazarou and Heinrich’s (2019) recent investigation of contemporary views of HM amongst the British general public, there was very little awareness of the lack of regulations. The majority of participants stated that they trust the supplier or rely on brand reputation. This is critical given the variability in quality observed in herbal products, and in one study around 25% of products containing turmeric root (*Curcuma longa*, Zingiberaceae) assessed were either poor quality or incorrect parts of the plant were used (Booker and Heinrich, 2016). The quality of over-the-counter herbal products is equally as important to assure along with ensuring herbal practice as a rigorously trained profession (Nissen, 2015).

Research shows that women are seeking advice on herbs from a range of sources. Some women gain knowledge through their upbringing, parents and friends, and perhaps due to a lack of trust in alternative practitioners (Holst et al., 2009). A similar point was made earlier in self-help decision-making in Ghanaian men living in the United Kingdom (Alidu and Grunfeld, 2020). This reluctance towards talking to a conventional HCP, and strong favour towards HMs for pregnancy based on recommendations from friends and family could be problematic in terms of circulating false information within groups (Holst et al., 2009). As discussed in the section on ethnic groups in the United Kingdom, there are potential herb-drug interactions which can occur without the advice of a qualified practitioner. These perceptions indicate that either better sources of herbal knowledge should be made available or compulsory regulation of herbal practitioners should be advocated to avoid members of the public relying on non-qualified individuals and advice from the internet. Further details regarding the participants demographic characteristics may have provided more context for these discussions.

Patients are also finding information via social media. A 2020 study focusing on patients’ knowledge of HM in Saudi Arabia concluded that the second most common method of acquiring knowledge was through social media platforms (Alqathama et al., 2020). Fitness, health and nutrition trends are sweeping social media, many that can positively contribute to a lifestyle and are promoted by legitimate experts in the field. However, this is not always the case. Influencers and celebrities advertise “detox”, “cleansing” and “natural” supplements that claim to promote fast weight-loss and diminish bloating. Many of these products contain herbs such as senna (*Senna alexandrina* Mill.), an effective laxative. Studies have shown that senna can cause diarrhoea, cramps and potentially potassium deficiency if chronically used, and is never advised to be taken for more than 10 days (Gardiner et al., 2000). These types of social media posts have come under scrutiny not only because unregulated products are often being promoted, but the knock-on effect that this type of promotion has on its audiences. By understanding what the most common sources of knowledge are, herbal practitioners, HCPs and policy makers can determine how to distribute professional knowledge.

#### 3.2.3 Sub-Theme Within Herbal Medicine and Gender: Herbalists Helping Women to Change Health Narratives

Understanding the perceptions of female herbalists themselves is often ignored in British ethnopharmacological research. Not only can this kind of study explore how herbalists’ practices differ from conventional HCPs, but they further investigate why women are drawn to becoming a practitioner. Understanding these experiences can bridge the gap between narratives of gender and perceptions of healthcare. Medical anthropologist Nina Nissen uses theoretical concepts of femininity to help construct narratives of HM usage in the United Kingdom. Using participant observation to focus on white middle-class female herbal practitioners and patients in her 2015 study, concepts surrounding the naturalness of HMs are associated with the idea of self-healing. This relates to holism and the contextualisation of the whole person. Patients have disclosed during consultations with their herbal practitioner that they have a lack of time for self-care and their self. Herbal practitioners have responded to this by urging women to “listen to your body” or to “listen to your heart” to understand and respond to their life experiences and facilitate self-discovery (Nissen, 2015). The consultation techniques are uncommon in conventional healthcare environments. Whilst all patients in this study turned to HM when they were ill, they also engaged with different views on their body and self-care (Nissen, 2015). This is a narrative that was identified in Warriner et al.’s research where HM was an attractive option due to the philosophical approach of interweaving physical and mental health (2014).

Drawing on understandings of embodiment, Nissen (2013) explores how women herbalists construct narratives about the body and healthcare. The participating herbalists saw their role as facilitators who paid attention to the patients’ stories and experiences. This included the sociocultural narratives surrounding a women’s health. One herbalist drew on the concept of constitutions as “a constellation of traits concerning body and self in health and illness, including physical appearance, personality, food and/or exercise preferences and physical and emotional reactions to stimuli.” By exploring old and new constitutions, herbalists offer something unique compared to other care professionals, fostering a sense of self-worth (Nissen, 2013). This aspect of herbal practice often comes under scrutiny, and a study of HM practitioners in Australia believed the medical community’s perception toward them was negative (Casey et al., 2008), partly thought to be due to the lack of evidence and variable quality of clinical studies on herbs where they do exist (Izzo et al., 2016).

By acknowledging that women’s gendered existence impacts their health, many users of HM turn to herbalists as they “*shape ideas and practices concerning bodies and health that seek to bridge the body, self, and society in the complex realities of women’s lives*”. Often these ideas originate from the herbalists’ own personal experiences, possibly a reaction against the reductionist nature of conventional healthcare in the UK, or in favour of the promotion of women’s autonomy in CAM (Nissen, 2013).

#### 3.2.4 Sub-Theme Within Herbal Medicine and Gender: The Male Body and Health

There are very few studies exploring herbal medicine use by men both in the UK and globally. The research that remotely touched upon it was explored in the context of the Afro-Caribbean population. Referring to Alidu and Grunfeld (2020)’s research focusing on Ghanaian men, it was concluded that men were reluctant to seek medical advice through fear of appearing weak, although this was not specifically in the context of HM use. In Nissen’s contextualisation of HM practitioners across England, the levels of male participation was low, with male practitioners and patients between 10%–20% of those involved (Nissen 2010).

The reality that men are less likely to seek help for their health is explored in many psychological, anthropological and socio-medical studies, especially in the context of mental health. A systematic review conducted by Yousaf et al. (2013) indicated that a range of psychological factors such as restricted emotional expression is a common barrier to men seeking professional help. In men with depression, there was a particular reluctance to seek help or to describe their emotions as men were not “supposed to” talk about such things (Yousaf et al., 2013). These fit well within traditional roles of masculinity and further stress would arise from trying to comply with the gender stereotype (Yousaf et al., 2013).

Nissen (2015) determined that there was a prominent emotional aspect to the practices of women herbalists where women patients seek treatments that allowed holistic and healthy expression. The lack of male patients may be due to traditional patterns of masculinity where the dismissal of tending to emotional needs pervades, thus leading to HM primarily responding to women’s needs (Nissen, 2010; Nissen, 2015). Further research focusing on a male perspective would provide insight as to what male self-care choices are and why, and it would be valuable to understand how men are positioned in what is seen as a female-orientated domain. This could build on research focusing on men’s attitudes towards self-care, as well as contemporary healthcare systems.

## 4 Conclusion

In the present study, ethnopharmacological research and mixed methodology emphasises the importance of a holistic lens, and that it is evident that there is more to HM utilisation than understanding the clinical efficacy of products. To establish a more culturally and socially sensitive medical system, a crucial issue reported in the literature reviewed, ethnographic research is imperative for understanding the cultural and socio-economic narratives of alternative healthcare practices. Loss of knowledge and culture was a major concern amongst the elderly in various ethnic groups in the UK, especially as the younger generations want to immerse themselves more into Westernised lifestyles. HMs sourced from across the world such as ashwagandha (*Withania somnifera* (L.) Dunal) from the Indian subcontinent, and baobab powder (*Adansonia digitata* L.) from Madagascar continue to be extremely popular in the United Kingdom. Such as the cases of yoga, meditation and veganism, the exotic element of HMs may indeed be appealing to the consumer, but how much is known about the historical and cultural context of these medicines? More importantly, is it important to the public (Gemar, 2020)? From a medical perspective, further training in HMs and phytotherapy will not only reduce the risk of drug-herb reactions and enable HCPs to relate better to diverse patients, but recent studies are recognising the shortage of new and effective pharmacological treatments. This could revert pharmaceutical companies back to HMs and other traditional medicines as potential sources of new treatments (Zahn et al., 2019).

A theme throughout the literature was how HM users acquire their knowledge. Information about HMs were mostly circulated around friends and family primarily due to the reluctance to discussing HMs with their conventional HCP, and because there is a lack of understanding or access to herbal practitioners. Although people turn to herbalists for the holistic and emotional aspects of their practice, herbalists in the UK are difficultly positioned as they aspire to practice HM within a “traditional” framework and integrate biomedical and evidence-based developments that result in the professionalisation of HM. It seems like the route to public legitimacy, especially in the UK, is rooted in determining the safety and efficacy of HM through clinical research that takes into consideration how herbs are used in a real-life setting. This would not only allow HCPs to make informed decisions regarding referring patients to a herbalist, but allow patients to explore HM without fear of judgement and misinformation. Further research focusing on the scepticism towards HM and herbal practitioners from a variety of groups is critical for the integration of HM within conventional medicine.

Research has consistently highlighted the negative experiences that women have encountered within conventional medical settings, which has led to them resorting to using HM. There is a clear lack of communication with HCPs regarding HMs which usually stems from a lack of knowledge or interest, and a strong feeling by patients that HCPs should undertake some training in herbalism to assist with conversations. It should be noted that whilst it may appear that the predominant users and practitioners of HM are white women, there is possible selection bias in the studies reviewed (Gokhale et al., 2003; Gentry-Maharaj et al., 2015). Other studies have shown that a large proportion of ethnic minority populations in the UK prefer HMs, particularly where women are the primary users and healers of the family (Bhamra et al., 2017).

The possibility to successfully integrate Western biomedicine and HM within formal healthcare systems has been demonstrated in many countries, such as China, Japan, Korea, India and Ghana (Zhang et al., 2011; Appiah et al., 2018). However, in some cases the same problems remain of a lack of trust in HM by conventional HCPs and concerns over efficacy and safety (Appiah et al., 2018), which can only be overcome through education and further research. In Ghana, HM treatment is subject to additional fees and it is likely that any future integration of HM into the healthcare system in the NHS would also be subject to extra cost. The complexity of herbs and herbal products means that it is not appropriate to use them simply as a substitute to a single pharmaceutical compound, however, connections with nature, tradition and a more holistic approach to health means that they remain a desirable option for many. Greater understanding of the broad range of benefits from HM by HCPs, combined with research that is designed to assess the way that HM is used in practice (rather than mimicking how herbs would be used if they were pharmaceutical drugs), will help to support safe and effective use of HM by the public.

To fully understand HM use in the UK from patients to healers, it is crucial to explore different cultural, social and economic narratives of health and healing. Future research needs to bridge gaps between anthropological ideologies of health and culture, to help HCPs in achieving the best results they can with increasingly diverse and curious patients. HMs do not exist in a healthcare vacuum. The reasons behind how and why they are used is as important as understanding the effects of herbs in the body. Future ethnopharmacology research needs to take this into account.

## References

[B1] Agbabiaka T. B. Spencer N. H. Khanom S. Goodman C. (2018). Prevalence of Drug-Herb and Drug-Supplement Interactions in Older Adults: a Cross-Sectional Survey. Br. J. Gen. Pract. 68, e711. Available at: https://bjgp.org/content/68/675/e711 (Accessed Apr 9, 2019). 10.3399/bjgp18X699101 30249608 PMC6145997

[B2] Akhtar N. Wong T. (2021). Hope for the Future: A Manifesto for the Next Ten Years, where Patients, People and Communities Come First. London, United Kingdom: College of Medicine, College of Medicine, 32–34. Available at: https://collegeofmedicine.org.uk/wp-content/uploads/2021/05/The-Manifesto-Final_2021.pdf .

[B3] Alidu L. Grunfeld E. A. (2020). 'What a Dog Will See and Kill, a Cat Will See and Ignore it': An Exploration of Health-Related Help-Seeking Among Older Ghanaian Men Residing in Ghana and the United Kingdom. Br. J. Health Psychol. 25 (4), 1102–1117. 10.1111/bjhp.12454 32656938

[B4] Alqathama A. Alluhiabi G. Baghdadi H. Aljahani L. Khan O. Jabal S. (2020). Herbal Medicine from the Perspective of Type II Diabetic Patients and Physicians: what Is the Relationship? BMC Complement. Med. Ther. 20 (1), 65. 10.1186/s12906-020-2854-4 32111222 PMC7076897

[B5] Appiah B. Amponsah I. K. Poudyal A. Mensah M. L. K. (2018). Identifying Strengths and Weaknesses of the Integration of Biomedical and Herbal Medicine Units in Ghana Using the WHO Health Systems Framework: a Qualitative Study. BMC Complement. Altern. Med. 18 (1), 286. 10.1186/s12906-018-2334-2 30348173 PMC6196414

[B6] Bhamra S. K. Slater A. Howard C. Heinrich M. Johnson M. R. D. (2019). Health Care Professionals' Personal and Professional Views of Herbal Medicines in the United Kingdom. Phytother. Res. 3333 (9), 2360–2368. Available at: https://onlinelibrary.wiley.com/doi/abs/10.1002/ptr.6418 . 10.1002/ptr.6418 31282109

[B7] Bhamra S. K. Slater A. Howard C. Johnson M. Heinrich M. (2017). The Use of Traditional Herbal Medicines Amongst South Asian Diasporic Communities in the UK. Phytother. Res. 31, 1786–1794. Available at: https://onlinelibrary.wiley.com/doi/abs/10.1002/ptr.5911 . 10.1002/ptr.5911 28905437

[B8] Bhopal R. S. (2012). The Quest for Culturally Sensitive Health-Care Systems in Scotland: Insights for a Multi-Ethnic Europe. J. Public Health (Oxf) 34 (1), 5–11. 10.1093/pubmed/fdr094 22267290

[B9] Booker A. Heinrich M. (2016). Value Chains of Botanical and Herbal Medicinal Products: A European Perspective. HerbalGram, 40–45. Available at: https://westminsterresearch.westminster.ac.uk/download/d9642f8bf6f954e94f26689510ab3df6558b10cc7ea71de5711b03f4418da290/2937989/Booker%26Heinrich-HerbalGram112-2016.pdf .

[B10] Braun V. Clarke V. (2006). Using Thematic Analysis in Psychology. Qual. Res. Psychol. 3, 77–101. Available at: https://www.tandfonline.com/doi/abs/10.1191/1478088706qp063oa . 10.1191/1478088706qp063oa

[B11] Casey M. Adams J. Sibbritt D. (2008). An Examination of the Clinical Practices and Perceptions of Professional Herbalists Providing Patient Care Concurrently with Conventional Medical Practice in Australia. Complement. Ther. Med. 16 (4), 228–232. 10.1016/j.ctim.2007.06.002 18638714

[B12] Ceuterick M. Vandebroek I. (2017). Identity in a Medicine Cabinet: Discursive Positions of Andean Migrants towards Their Use of Herbal Remedies in the United Kingdom. Soc. Sci. Med. 177, 43–51. 10.1016/j.socscimed.2017.01.026 28157568

[B13] Ceuterick M. Vandebroek I. Torry B. Pieroni A. (2008). Cross-cultural Adaptation in Urban Ethnobotany: The Colombian Folk Pharmacopoeia in London. J. Ethnopharmacol. 120 (3), 342–359. 10.1016/j.jep.2008.09.004 18852036

[B14] Ceuterick M. Vandebroek I. Torry B. Pieroni A. (2007). “The Use of Home Remedies for Healthcare and Well-Being by Spanish-Speaking Latino Migrants in London: Reflections on Acculturation,” in Traveling Cultures and Plants. The Ethnobiology and Ethnopharmacy of Migrations (New York- Oxford: Berghahn Books), 145

[B15] Fitzpatrick K. Berbesque J. C. (2018). Hunter‐Gatherer Models in Human Evolution. Int. Encycl. Anthropol., 1–10. 10.1002/9781118924396.wbiea2051

[B16] Flower A. Winters D. Bishop F. L. Lewith G. (2015). The Challenges of Treating Women with Recurrent Urinary Tract Infections in Primary Care: a Qualitative Study of GPs' Experiences of Conventional Management and Their Attitudes towards Possible Herbal Options. Prim. Health Care Res. Dev. 16 (06), 597–606. 10.1017/S1463423615000201 25772398

[B17] Frost R. Bhamra S. K. Pendry B. Heinrich M. (2021). COVID-19 and Herbal Practice: A United Kingdom Practitioner Survey. Adv. Integr. Med. 8 (4), 256–260. 10.1016/j.aimed.2021.09.003 34888138 PMC8452456

[B18] Gardiner P. Conboy L. A. Kernper K. J. (2000). Herbs and Adolescent Girls: Avoiding the Hazards. Contemp. Paediatr. 17 (3), 133–154.

[B19] Gemar A. (2020). Cultural Capital and Emerging Culture: the Case of Meditation, Yoga, and Vegetarianism in the UK. Leisure/Loisir 44 (1), 1–26. 10.1080/14927713.2020.1745671

[B20] Gentry-Maharaj A. Karpinskyj C. Glazer C. Burnell M. Ryan A. Fraser L. (2015). Use and Perceived Efficacy of Complementary and Alternative Medicines after Discontinuation of Hormone Therapy: a Nested United Kingdom Collaborative Trial of Ovarian Cancer Screening Cohort Study. Menopause 22 (4), 384–390. 10.1097/GME.0000000000000330 25290539 PMC4470524

[B21] Gokhale L. Sturdee D. W. Parsons A. D. (2003). The Use of Food Supplements Among Women Attending Menopause Clinics in the West Midlands. J. Br. Menopause Soc. 9 (1), 32–35. 10.1258/136218003100321991 12804311

[B22] Hetland G. Johnson E. Bernardshaw S. V. Grinde B. (2021). Can Medicinal Mushrooms Have Prophylactic or Therapeutic Effect against COVID-19 and its Pneumonic Superinfection and Complicating Inflammation? Scand. J. Immunol. 93 (1), e12937. 10.1111/sji.12937 32657436 PMC7404338

[B23] Holst L. Wright D. Nordeng H. Haavik S. (2009). Use of Herbal Preparations during Pregnancy: Focus Group Discussion Among Expectant Mothers Attending a Hospital Antenatal Clinic in Norwich, UK. Complement. Ther. Clin. Pract. 15, 225–229. Available at: https://www.sciencedirect.com/science/article/abs/pii/S1744388109000516 . 10.1016/j.ctcp.2009.04.001 19880086

[B24] Howard P. (2001). Women in the Plant World: The Significance of Women and Gender Bias for Biodiversity Conservation. International Union for Conservation of Nature, 1–28. Available at: https://portals.iucn.org/library/sites/library/files/documents/Rep-2001-028.pdf .

[B25] IPSOS Mori (2008). Public Perceptions of Herbal Medicines: General Public Qualitative and Quantitative Research. Available at: https://www.ipsos.com/sites/default/files/migrations/en-uk/files/Assets/Docs/Polls/public-perceptions-of-herbal-medicines-report.pdf.

[B26] Izzo A. A. Hoon-Kim S. Radhakrishnan R. Williamson E. M. (2016). A Critical Approach to Evaluating Clinical Efficacy, Adverse Events and Drug Interactions of Herbal Remedies. Phytother. Res. 30 (5), 691–700. Available at: https://onlinelibrary.wiley.com/doi/abs/10.1002/ptr.5591 . 10.1002/ptr.5591 26887532

[B27] Jäger A. (2015). “Medicinal Plant Research: A Reflection on Translational Tasks,” in Ethnopharmacology (UK: John Wiley & Sons), 11–16.

[B28] Jeffery L. Rotter R. (2016). Sustenance, Nourishment, and Cultivation: Plants as Living Cultural Heritage for Dispersed Chagossians in Mauritius, Seychelles, and the UK. J. R. Anthropol. Inst. 22 (2), 296–313. 10.1111/1467-9655.12402

[B29] Kumar K. Greenfield S. Raza K. Gill P. Stack R. (2016). Understanding Adherence-Related Beliefs about Medicine Amongst Patients of South Asian Origin with Diabetes and Cardiovascular Disease Patients: a Qualitative Synthesis. BMC Endocr. Disord. 16, 24. Available at: https://www.ncbi.nlm.nih.gov/pmc/articles/PMC4880880/ . 10.1186/s12902-016-0103-0 27230479 PMC4880880

[B30] Lazarou R. Heinrich M. (2019). Herbal Medicine: Who Cares? the Changing Views on Medicinal Plants and Their Roles in British Lifestyle. Phytotherapy Res. 33 (9), 2409–2420. Available at: https://onlinelibrary.wiley.com/doi/abs/10.1002/ptr.6431 . 10.1002/ptr.6431 31313391

[B31] Leonti M. Casu L. (2013). Traditional Medicines and Globalization: Current and Future Perspectives in Ethnopharmacology. Front. Pharmacol. 4, 92. 10.3389/fphar.2013.00092 23898296 PMC3722488

[B32] Leonti M. Weckerle W. (2015). “Quantitative and Comparative Methods in Ethnopharmacology,” in Ethnopharmacology (John Wiley & Sons). 10.1002/9781118930717.ch4

[B33] Little C. V. (2009). Simply Because it Works Better: Exploring Motives for the Use of Medical Herbalism in Contemporary U.K. Health Care. Complement. Ther. Med. 17 (5-6), 300–308. 10.1016/j.ctim.2009.08.001 19942110

[B34] Nissen N. (2015). Naturalness as an Ethical Stance: Idea(l)s and Practices of Care in Western Herbal Medicine in the UK. Anthropol. Med. 22 (2), 162–176. 10.1080/13648470.2015.1043789 26001272

[B35] Nissen N. (2010). Practitioners of Western Herbal Medicine and Their Practice in the UK: Beginning to Sketch the Profession. Complement. Ther. Clin. Pract. 16, 181–186. Available at: https://www.sciencedirect.com/science/article/abs/pii/S1744388110000484 10.1016/j.ctcp.2010.06.001 20920799

[B36] Nissen N. (2013). Women's Bodies and Women's Lives in Western Herbal Medicine in the UK. Med. Anthropol. 32, 75–91. Available at: https://www.tandfonline.com/doi/abs/10.1080/01459740.2012.674079. 10.1080/01459740.2012.674079 23206176

[B37] Petrovska B. B. (2012). Historical Review of Medicinal Plants' Usage. Pharmacogn. Rev. 6 (11), 1–5. 10.4103/0973-7847.95849 22654398 PMC3358962

[B38] Pieroni A. Sheikh Q. Z. Ali W. Torry B. (2008). Traditional Medicines Used by Pakistani Migrants from Mirpur Living in Bradford, Northern England. Complement. Ther. Med. 16, 81–86. Available at: https://www.sciencedirect.com/science/article/abs/pii/S0965229907000398 . 10.1016/j.ctim.2007.03.005 18514909

[B39] Pieroni A. Torry B. (2007). Does the Taste Matter? Taste and Medicinal Perceptions Associated with Five Selected Herbal Drugs Among Three Ethnic Groups in West Yorkshire, Northern England. J. Ethnobiol. Ethnomed 3 (1), 21. 10.1186/1746-4269-3-21 17475019 PMC1872019

[B40] Porqueddu T. (2017). Herbal Medicines for Diabetes Control Among Indian and Pakistani Migrants with Diabetes. Anthropol. Med. 24 (1), 17–31. 10.1080/13648470.2016.1249338 28292208

[B41] Ritchie M. R. (2007). Use of Herbal Supplements and Nutritional Supplements in the UK: what Do We Know about Their Pattern of Usage? Proc. Nutr. Soc. 66, 479–482. Available at: https://www.cambridge.org/core/journals/proceedings-of-the-nutrition-society/article/use-of-herbal-supplements-and-nutritional-supplements-in-the-uk-what-do-we-know-about-their-pattern-of-usage/2261131B761B00F4AE8F7FC5B63B0B64 . 10.1017/S0029665107005794 17961268

[B42] Sandhu D. S. Heinrich M. (2005). The Use of Health Foods, Spices and Other Botanicals in the Sikh Community in London. Phytother. Res. 19 (7), 633–642. 10.1002/ptr.1714 16161027

[B43] Shaw A. Noble A. Salisbury C. Sharp D. Thompson E. Peters T. J. (2008). Predictors of Complementary Therapy Use Among Asthma Patients: Results of a Primary Care Survey. Health Soc. Care Community 16 (2), 155–164. 10.1111/j.1365-2524.2007.00738.x 18290981

[B44] Sodhi M. S. Tang C. S. (2017). Corporate Social Sustainability in Supply Chains: A Thematic Analysis of the Literature. Int. J. Production Res. 56 (1–2), 882–901. 10.1080/00207543.2017.1388934

[B45] Toms C. Cahill F. George G. Van Hemelrijck M. (2016). Research Engagement Among Black Men with Prostate Cancer. ecancermedicalscience 10, 695. Available at:https://www.ncbi.nlm.nih.gov/pmc/articles/PMC5215282/ . 10.3332/ecancer.2016.695 28101138 PMC5215282

[B46] Tupper K. W. (2009). Ayahuasca Healing beyond the Amazon: the Globalization of a Traditional Indigenous Entheogenic Practice. Glob. Netw. 9 (1), 117–136. 10.1111/j.1471-0374.2009.00245.x

[B47] Vickers K. A. Jolly K. B. Greenfield S. M. (2006). Herbal Medicine: Women’s Views, Knowledge and Interaction with Doctors: a Qualitative Study. BMC Complementary Altern. Med. 6, 40. Available at: https://bmccomplementalternmed.biomedcentral.com/articles/10.1186/1472-6882-6-40 . 10.1186/1472-6882-6-40 PMC1702550 17156416

[B48] Waldstein A. (2006). Mexican Migrant Ethnopharmacology: Pharmacopoeia, Classification of Medicines and Explanations of Efficacy. J. Ethnopharmacol. 108 (2), 299–310. 10.1016/j.jep.2006.07.011 16934952

[B49] Walker D. (2015). Report on the Regulation of Herbal Medicines and Practitioners. Gov.uk. Medicines and Healthcare products Regulatory Agency. [online]Available at: https://www.gov.uk/government/publications/advice-on-regulating-herbal-medicines-and-practitioners .

[B50] Warriner S. Bryan K. Brown A. M. (2014). Women's Attitude towards the Use of Complementary and Alternative Medicines (CAM) in Pregnancy. Midwifery 30 (1), 138–143. 10.1016/j.midw.2013.03.004 23631887

[B51] Yousaf O. Grunfeld E. A. Hunter M. S. (2013). A Systematic Review of the Factors Associated with Delays in Medical and Psychological Help-Seeking Among Men. Health Psychol. Rev. 9 (2), 264–276. 10.1080/17437199.2013.840954 26209212

[B52] Zahn R. Perry N. Perry E. Mukaetova-Ladinska E. B. (2019). Use of Herbal Medicines: Pilot Survey of UK Users' Views. Complement. Ther. Med. 44, 83–90. Available at:https://www.sciencedirect.com/science/article/abs/pii/S0965229919300214 . 10.1016/j.ctim.2019.02.007 31126579

[B53] Zhang A. L. Changli Xue C. Fong H. H. S. (2011). “Integration of Herbal Medicine into Evidence-Based Clinical Practice: Current Status and Issues,” in Herbal Medicine: Biomolecular and Clinical Aspects. Editors Benzie I. F. F. Wachtel-Galor S.. 2nd edition (Boca Raton (FL): CRC Press/Taylor & Francis). Chapter 22. 22593929

