# Supplementary material for: Narratives of Herbal Medicine Utilisation in the United Kingdom: Scoping Literature Review
Source: Front Pharmacol. 2022 Aug 25;13:886574. doi: 10.3389/fphar.2022.886574 (PMC9452627; doi:10.3389/fphar.2022.886574)
Supplement: Supplementary file 1 [file Table1.pdf]

Table 1: Summary of studies identified through the literature search

Authors and Date	Study Title	Population	Objectives	Outcomes	Study No.
Agbabiaka, et al., 2018	Prevalence of drug-herb and drug-supplement interactions in older adults: A cross-sectional survey	76 females and 71 males over 65 years	To investigate the ways that older adults are concurrently using prescription drugs with HMs and dietary supplements.	Females were more likely than males to use prescription drugs alongside HMs and supplements, and the use of dietary supplements with prescription drugs was more common than use of HMs.	1
Akinlua, et al., 2107	Beliefs about hypertension among Nigerian immigrants to the United Kingdom: A qualitative study	27 Nigerian migrants	To explore beliefs about hypertension among Nigerian immigrants in West London.	Differing beliefs amongst participants about hypertension, its causes and its treatment took into account orthodox and culturally framed ideas.	2
Alidu and Grunfeld, 2020	'What a dog will see and kill, a cat will see and ignore it': An exploration of health-related help-seeking among older Ghanaian men residing in Ghana and the United Kingdom	26 Ghanaian men over 50 years old in the UK and in Ghana	To explore approaches to managing symptoms and health-related help-seeking among older Ghanaian men in Kumasi and in London.	Ghanaian men living in Ghana and the UK differed in their help-seeking experiences. The factors that prevented help-seeking were financial or due to masculinity norms. Multiple approaches to how health and illness are managed, faith-based beliefs about illness, as well as perceptions about formal health services in both countries were also themes impacting help-seeking behaviour.	3
Alshawish, et al., 2013	Investigating access to and use of maternity health-care services in the UK by Palestinian women	22 Palestinian women	To investigate how Palestinian women use health services in the UK.	Culturally appropriate care is needed for BME groups. This can be achieved through training for HCPs into culture, ethnicity and religion, as well as interpretation services.	4
Barnes, et al. 2002	Different standards for reporting ADRs to herbal remedies and conventional OTC medicines: face-to-face interviews with 515 users of	515 herbal remedy users	To determine whether HM users report ADRs to herbal remedies differently from ADRs to conventional OTC medicines.	Those taking HMs are less likely to report an ADR associated with a herbal remedy. However, a high number of consumers would also not report an ADR from a conventional OTC medicine.	5

	herbal remedies.				
Bhamra, et al. 2017	The use of traditional herbal medicines amongst South Asian diasporic communities in the UK.	192 South Asian adults	To explore traditional HM knowledge and how it is transmitted by South Asian communities in the UK.	South Asian communities in the UK use both traditional and conventional medicines. Knowledge of these traditional HMs is verbally passed on from generation to generation. It can be considered that HMs still have an important role in health.	6
Bhamra, et al. 2019	Health care professionals' personal and professional views of herbal medicines in the United Kingdom.	93 healthcare professionals including doctors, surgeons, nurses, dentists, pharmacists, optometrists, physiotherapists and herbal medicine practitioners	To get an insight into HCPs knowledge of HMs via their personal and professional opinions and their perception of the safety and efficacy of HMs.	38% of HCPs personally used HMs. Having a positive personal experience of HMs meant that HCPs were more likely to recommend them to patients. HCPs felt unable to advise patients on safe use of HMs due to a lack of knowledge and training.	7
Canter and Ernst, 2004	Herbal Supplement Use by Persons Aged Over 50 years in Britain.	271 participants over the age of 50	To explore the Herbal Products used by older people in the UK and potential for interactions with other drugs	Older people who use HMs in the UK often use multiple herbs alongside several nutritional supplements. This is rarely reported to doctors, which places people at risk of negative herb-drug reactions	8
Ceuterick and Vandebroek, 2017	Identity in a medicine cabinet: Discursive positions of Andean migrants towards their use of herbal remedies in the United Kingdom.	40 Bolivian and Peruvian Migrants	To understand how Andean migrants explain their use of herbal remedies in the UK and how is language about their preference for herbal remedies used to construct identities	The way that migrants explained their use of HMs revealed that it could be framed as a tradition, health-conscious consumer choice, or coping strategy. Understanding the various different reasons that inform use of traditional HMs will enable HCPs to help patients make decisions about their health.	9

Ceuterick, et al., 2007	The Use of Home Remedies for Healthcare and Well-Being by Spanish-Speaking Latino Migrants in London: Reflections on Acculturation.	35 Andean immigrant	To explore the plants that are used for health care by Latino immigrants in London, and the influence of migration on these medical practices	HMs are used by Latino immigrants in home remedies for minor ailments. They can be considered medicinal herbs for preventative or curative health, functional foods, ritual plants/remedies, or cosmetic home remedies.	10
Ceuterick, et al., 2008	Cross-cultural adaptation in urban ethnobotany: The Colombian folk pharmacopoeia in London	23 Colombians	To illustrate the process of cross-cultural adaptation and change in medicinal plant use by Colombians in London through a review of continued, changed and possible new medicinal plant uses	There is cross-cultural adaptation within Colombian communities in London, in which some traditional uses are maintained (to hold on to identity, or a reflection of plant efficacy), some are lost (deculturation) and some new remedies are adopted (acculturation towards the new society).	11
Flower, et al., 2015	The challenges of treating women with recurrent urinary tract infections in primary care: a qualitative study of GPs' experiences of conventional management and their attitudes towards possible herbal options.	15 GPs in practice from 3 to 31 years	To explore GPs' experiences of managing RUTIs in primary care in general and their views on the possible role of herbal medicines. The primary aim was to better understand, from GPs' perspectives, the challenges in managing RUTIs. The secondary aim was to identify possible opportunities and barriers for using herbal medicines, including CHM, in this context.	Participants who were open to the use of HMs wanted to see more research into effectiveness and safety (particularly potential herb-drug interactions), as well as practitioner regulation and better quality control.	12
Freymann, et al. 2006	Knowledge and Use of Complementary and Alternative Medicine among British Undergraduate Pharmacy Students.	264 undergraduate students at the London School of Pharmacy	To understand the use of herbal medicinal products, medicinal plants and other natural remedies among a culturally diverse undergraduate student population at The School of Pharmacy, University of London.	The participants had widespread knowledge about and interest in CAM. They were also users of CAM. Understanding use of CAM among ethnic minorities and HCPs may be helpful for the treatment of acute minor disease, as well as chronic conditions.	13

Gentry-Maharaj, et al., 2015	Use and perceived efficacy of complementary and alternative medicines after discontinuation of hormone therapy	10,607 postmenopausal women aged 50-65	The use and perceived benefits of CAMs for relief of VMS in women who discontinued hormone therapy after the Women's Health Initiative report, using a nested cohort design within the United Kingdom Collaborative Trial of Ovarian Cancer Screening.	OTC medicines were more commonly used, however, behavioural/lifestyle approaches to health provided better relief of VMS in these women. Better evidence-based information is needed for people to make decisions about the use of CAM for VMS.	14
Gokhale, et al. 2003	The use of food supplements among women attending menopause clinics in the West Midlands	340 women	To estimate the extent of use of OTC food supplements in women attending menopause clinics in the West Midlands	Food supplement use is widespread, particularly in those taking HRT. Users report benefiting from a feeling of wellbeing. Participants also falsely believed that herbal or natural products were always safe.	15
Hallala and Phiri, 2020	Executive summary of public perceptions about alternative medicine in Angel, London.	100 participants based in Angel, London	To identify the public perceptions about alternative medicine in Angel, London, including what qualifications and qualities the public expect from CAM practitioners.	CAM modalities mentioned included Chinese medicine, acupuncture, massage, homeopathy, herbal teas and cosmetic products, as well as traditional medicine from their own country. 53% of participants would use CAM in the future. 34% of participants expected practitioners to have professional qualifications.	17
Harrison, et al. 2004	Who and how many people are taking herbal supplements? A survey of 21923 adults.	15,465 adults	Exploring the number and characteristics of adults taking herbal supplements and the relationship of this with other health and lifestyle factors,	More than one in ten adults were taking herbal supplements, with evening primrose oil the most common supplement, used mainly by women. Individual characteristics such as age, sex, ethnicity and social class influenced the use of herbal supplements. Participants were using supplements in addition to rather than instead of conventional care.	18

Holst, et al. 2009	Use of herbal preparations during pregnancy: Focus group discussion among expectant mothers attending a hospital antenatal clinic in Norwich, UK.	6 women	To investigate why women choose to use HMs in pregnancy.	The study identified that participants rely on family and friends for information, and felt that HM were safe, yet understudied. There was a theme of taking HM being "underground" and participants wanted the NHS to be more open minded.	19
Jeffery and Rotter, 2016	Sustenance, nourishment, and cultivation: plants as living cultural heritage for dispersed Chagossians in Mauritius, Seychelles, and the UK.	Chagossian migrants	To examine the meaning that plants have for displaced Chagossian communities in various locations.	Plants provide a living cultural heritage for these communities suffering loss, dislocation and dispersal around the world.	20
Lazarou and Heinrich, 2019	Herbal medicine: Who cares? The changing views on medicinal plants and their roles in British lifestyle	408 visitors of the Eden Project	To explore how the sampled public use and source HMs, as well as assessing their knowledge. How this connects to changing lifestyles in the UK is also addressed.	Both men and women had similar attitudes towards HM, with women generally being more positive. Those in the 36-55 year-old age group were most likely to use HM. Popular reasons for HM use included a desire to use HM for minor conditions rather than pharmaceuticals, as they are more natural and have less side effects. Popular reasons to use HM included overall wellbeing, sleep, immunity and digestion.	21
Little, 2009	Simply because it works better: Exploring motives for the use of medical herbalism in contemporary U.K. health care.	19 adults who were consulting with registered medical herbalists	To investigate why people in the UK consult with herbalists.	Participants consulted herbalists because they felt it worked and had greater consistency with their understanding about health and illness than conventional medicine.	22

Nissen, 2010	Practitioners of Western herbal medicine and their practice in the UK: Beginning to sketch the profession	55 Western Herbal Medicine practitioners	To profile the profession of Western herbal medicine in the UK	Herbal medicine practitioners are interested in natural healing and want to help others. Western HM is suitable for women's health needs. A typical practitioner is aged 41-50, white, female and works part-time. There are tensions in HM surrounding a 'traditional' framework of healthcare and evidence-based developments.	23
Nissen, 2013	Women's Bodies and Women's Lives in Western Herbal Medicine in the UK.	6 female Western herbal medicine practitioners	To examine how ideas about the body, health, and health care are constructed by women herbalists, and how women patients integrate these ideas and related practices concerning the care for body and self into their everyday lives.	Women use HM and related narratives of health to assert values of self and justify self-care. Gender and health are intertwined, so that in addition to being holistic, Western HM is politicised and both confronts and fulfils traditional gender roles. Feminine values such as caring are supported.	24
Nissen, 2015	Naturalness as an ethical stance: idea(l)s and practices of care in western herbal medicine in the UK.	6 female Western herbal medicine practitioners and 9 patients	To examine ideas of naturalness in relation to Western HM.	Herbalists and their patients are linked by ideas about the goodness of nature, its connection with holistic care, and a shared humanity with the organic world.	25
Pieroni, et al., 2008	Traditional medicines used by Pakistani migrants from Mirpur living in Bradford, Northern England	37 members of the Pakistani community from Mirpur living in Bradford	To record traditional medicines and foods known and still used among Pakistani migrants from Mirpur in Bradford. How this is changing in different generations moving from Pakistan to Bradford is also assessed.	Traditional medicines are still popular amongst the migrants, with 2/3 of interviewees preferring HM to conventional medicine. Participants listed 56 remedies that are still used (more than half of which were foods). Younger generations and those who have been in Bradford longer had less knowledge.	26
Pieroni and Torry, 2007	Does the taste matter? Taste and medicinal perceptions associated with five selected herbal drugs among three	254 participants belonging to Gujarati, Kashmiri and English ethnic groups	To explore whether taste retains an importance in determining medicinal perceptions of botanicals.	Traditional knowledge is most prevalent in Kashmiri groups and least in the English group. Links between the perception of taste and medicinal use of a plant depend on both human	27

	ethnic groups in West Yorkshire, Northern England			physiology and individual experiences and culture.	
Porqueddu, 2017	Herbal medicines for diabetes control among Indian and Pakistani migrants with diabetes	21 Indian and Pakistani migrants in Edinburgh	To explore Indians' and Pakistanis' use of herbal medications for diabetes control	Participants used non-allopathic remedies so that they could reduce allopathic medication and thus avoid side effects. HM was thought to cure the cause of the disease rather than just treat symptoms. Participants eventually replaced allopathic medication without consulting their doctor.	28
Robinson, et al., 2008	Complementary medicine use in multi-ethnic paediatric outpatients.	189 parents	Investigating use of CAM for children with chronic disease.	CAM use was 37% and related to whether parents used CAM themselves, and their health and educational status. However, ethnic group was not an indicator of CAM use. Parents decided to use CAM due to word of mouth information and 51% stated that their main source of information was family and friends. Most commonly used CAM therapies were HM and homeopathy.	29
Robinson and Lorenc, 2010	Responding to patient demand: community pharmacists and herbal and nutritional products for children	5 Pharmacists and 17 other staff	Investigating attitudes and behaviour of pharmacists with regard to HM for children.	Participants noticed that there was an increasing demand for herbal and nutritional products for children. They were open to the use of these products and felt able to give advice, but wanted better knowledge to maintain professionalism.	30
Rochelle and Marks, 2010	Health Behaviours and Use of Traditional Chinese Medicine Among the British Chinese	186 members of the British Chinese community	To explore whether the British Chinese community had a preference for traditional Chinese medicine or Western medicine.	Most participants used Western medicine due to cost and effectiveness. Speaking a Chinese dialect as their first language, a sense of cultural superiority and a belief that health behaviour is influenced by cultural and religious values were all associated with use	31

				of Chinese traditional medicine.	
Sandhu and Heinrich, 2005	The use of health foods, spices and other botanicals in the Sikh community in London.	84 members of the Sikh community	To explore the use of botanical and traditional medicines in the Sikh community in London	Of the 42 species identified as being used by the Sikh community in London, the most common were foods such as onion, garlic, cayenne pepper, cinnamon, lemon, fennel, ginger and cardamom. Traditional Sikh medicine was important to the respondents and CAM use may help for the management of minor ailments and chronic disease.	32
Scott, et al., 2005	Use of complementary and alternative medicine in patients with cancer: A UK survey.	127 patients with cancer	To assess the use of CAM therapies by patients with cancer	CAM use is increasing in the UK, with the most commonly used therapies being meditation/relaxation and medicinal teas. 29% of the sample used CAM, and most found their information from media (57), friends (57) and family (31). Availability and cultural differences are likely to account for differences in use between Europe and the UK.	33
Smith, et al., 2004	Co-ingestion of herbal medicines and warfarin	1360 patients using warfarin	A postal study was used to estimate how many patients were using warfarin alongside HMs.	Of the 1360 respondents, 19.2% were taking one or more HM or homeopathic treatment, with 8.8% of them taking a therapy that may interfere with warfarin. 28.3% of respondents recognised that HM may or would interfere with drugs.	34
Toms, et al., 2016	Research engagement among black men with prostate cancer.	16 black males with prostate cancer	To explore why black males with prostate cancer are less likely to take part in clinical trials	In addition to the expected barriers of mistrust and lack of understanding, belief in and use of HMs impacted participation in studies.	35
Vickers, et al., 2006	Herbal medicine: women's views, knowledge and interaction with doctors: a	18 white British women	To investigate women's views of HM safety, herb-drug interaction and whether they communicated with their doctor about this.	Most women did not tell their doctor about use of HM. Reasons for this include that the doctor did not ask, the women didn't feel it was necessary or they feared the response from the doctor.	36

	qualitative study.			Any adverse events from HMs were not reported to the doctors.	
Waldstein, 2019	Smoking as Communication in Rastafari: Reasonings with 'Professional' Smokers and 'Plant Teachers.'	Rastafari smokers	To explore the idea of smoking cannabis as a communication between plants and smokers.	'Professional' smokers undertake rituals so that cannabis takes on a role of 'plant teacher'. In this way both smoker and plant are subjects interacting.	37
Warriner, et al., 2014	Women's attitude towards the use of complementary and alternative medicines (CAM) in pregnancy.	10 pregnant women	To explore OTC and CAM use in pregnant women.	Participants focused on the benefits of CAM and contrasted this with the disadvantages of conventional medicine. CAM was considered a holistic approach to health that the women had control over and they did not inform their doctors or midwives about this use.	38
Yöney, et al., 2009	Ethnopharmacy of Turkish-speaking Cypriots in greater London	87 members of the Turkish-speaking Cypriots	To explore the medicinal plant use by Turkish-speaking Cypriots in London	Of the 85 different species used, most were from Turkish-Cypriot traditional medicine, however, there were also plants from the UK and Western HM. Participants were concerned about the loss of knowledge due to young people being less likely to use medicinal plants.	39
Zahn, et al., 2019	Use of herbal medicines: Pilot survey of UK users' views.	157 medicinal plant users	To explore how and why people use HMs and where they find information about them.	Participants used HMs as they believed in their efficacy. They were more likely to find out information from books (57%), the internet (53%) and friends or neighbours (51%), than from HCPs (42%). Around half of the doctors that participants spoke to were positive about HM use. Further training for HCPs will assist with patient-centred care and so that people can seek out herbal solutions to health	40

				problems confidently and supported by their doctor.	

Key: ADR, adverse drug reaction; CAM, complementary and alternative medicine; BME, black and minority ethnic; HCP, healthcare professional; HM, herbal medicine; OTC, over-the-counter; VMS, vasomotor symptoms.

Table 2: Allocation of studies to numerical codes for different study aims, data collection methods, locations and study populations

Study no	Study Aim	Location	Data Collection	Population	Sub-Group	Year Published
**1**	2<br>5	1<br>9	1	2	3	2018
**2**	6	1	3	5	12	2017
				2	3	
**3**	12<br>13	1	3	5<br>1<br>2	12<br>1<br>3	2020
**4**	7	3	3	5<br>1	20<br>2	2013
**5**	8	3<br>6<br>4<br>10<br>11<br>12	2	6		2002
**6**	1<br>2	13<br>5	1	5	13	2017
**7**	1<br>2	2	1	4	9	2019
**8**	2<br>5	7	1	2	3	2004
**9**	1	1	3	5	14	2017
**10**	1<br>2	1	3<br>4	5	14	2007
**11**	7<br>1<br>2	1	3<br>5	5	14	2008
**12**	3	14	3	4<br>3	9<br>8.5	2015
**13**	1	1	1<br>3	4	11	2006

**14**	3	2	1	1<br>3	2<br>5	2015
**15**	3	5<br>15	1	1<br>3	2<br>5	2003
**16**	2		6	3	7	2009
**17**	1	1	1	6		2020
**18**	2	3	1	6		2004
**19**	1<br>3	16	7	1<br>3	2<br>6	2009
**20**	13<br>1	17<br>1<br>3	3	5	17	2016
**21**	1<br>2	2	1<br>3	6		2019
**22**	2	2	3	7		2009
	1					2009
**23**	4	1<br>18<br>19<br>8	1	4	10	2010
**24**	4	2	3<br>4	1<br>4	2<br>10	2013
**25**	4	2	3<br>4	4<br>7	10	2015
**26**	1<br>2	4	3<br>7	5	13	2008
**27**	14<br>9	4	5<br>1<br>5	5	13<br>19	2007
**28**	3<br>14	20	3<br>4<br>7	3<br>5	8.75<br>13	2017
**29**	3	1	1	8		2008
**30**	1	1	3<br>8	4	9	2010
**31**	1<br>2	4<br>21<br>3<br>5<br>10<br>6	1	5	15	2010

		1<br>22				
**32**	1<br>2	1	5<br>3	5	13	2005
**33**	1<br>2	2	1	3	7	2005
**34**	1<br>5	8<br>7	1	3	8	2004
**35**	10	1	7	1<br>3<br>5	1<br>7<br>12	2016
**36**	1	23	3	1	2	2006
	7					
**37**	11	1<br>8<br>24	3<br>4	5	16	2019
**38**	3	2	1<br>3	3	6	2014
**39**	1<br>2	1	3	5	18	2009
**40**	1<br>2	2	1	6		2019

Key to numerical codes for study aims, indicating the total number of studies allocated to each aim

Code	Study Aim	Total
1	Perception of HM	20
2	Use of HM	16
3	Perceptions of HM for Health Conditions	7
4	Perception of HM Practitioners	3
5	Herb-Drug Interaction	3
6	Perception of Health Conditions	1
7	Access to Health Care	3
8	ADR Reactions	1
9	Chemsensory Perceptions	1
10	Research Engagement	1
11	Spiritual Uses	1
12	Perceptions of Health Care	1
13	Cross Country Comparison	2
14	Cross Ethnicity Comparison	2

Key to numerical codes for locations, indicating the total number of studies allocated to each location

Code	Location	Total
1	London	17
2	Unspecified	9
3	Greater Manchester	5
4	West Yorkshire	4
5	West Midlands	3
6	Tyne and Wear	2
7	Devon	2
8	Somerset	3
9	Essex	1
10	Buckinghamshire	2
11	South Glamorgan	1
12	West Glamorgan	1
13	Leicester	1
14	Hampshire	1
15	Warwickshire	1
16	Norfolk	1
17	West Sussex	1
18	Surrey	1
19	Lancashire	1
20	Lothian	1
21	Meyride	1
22	South Yorkshire	1
23	Cheshire	1
24	Bristol	1

Key to numerical codes for data collection methods, indicating the total number of studies allocated to each method

Code	Data Collection	Total
1	Survey/Questionnaire	18
2	Structured Interview	1
3	Semi-Structured Interview	21
4	Participant Observation	5
5	Free-Listing	4
6	Systematic Review	1
7	Focus Group	4
8	Diary Recording	1

Key to numerical codes for populations, indicating the total number of studies allocated to each population and population sub-group

Code	Population	Total
1	Gender	8
2	Age	4
3	Health Conditions	10
4	Health Care Professional	7
5	Ethnic/Religious Groups	16
6	General Public	5
7	HM Users	2

Code	Sub-Group	Total
1	Male	2
2	Female	6
3	Older	4
4	Younger	0
5	Post-Menopause	2
6	Pregnancy	2
7	Cancer	3
8	Warfarin Users	1
8.5	UTIs	1
8.75	Diabetes	1
9	Conventional HCP	3
10	HM Practitioner	3
11	HCP Students	1
12	Afro-Caribbean	3
13	South Asian	5
14	Andean	3
15	Chinese British	1
16	Rastafarian	1
17	Chagossian	1
18	Cypriot	1
19	British	1
20	Palestinian	1
